# Sex differences in gastric cancer mutational burden reflect MLH1-associated epigenetic regulation

**DOI:** 10.1186/s12885-026-16327-1

**Published:** 2026-06-15

**Authors:** Noa Klein, Dorit Shweiki

**Affiliations:** Bioinformatics Program, School of Computer Science, The Academic College of Tel Aviv-Yaffo, Tel Aviv, Israel

**Keywords:** Sex differences, Gastric cancer, Tumor mutational burden, MLH1, DNA repair, DNA methylation, Immunotherapy, Biomarkers

## Abstract

**Background:**

Tumor mutational burden (TMB) is widely used as a biomarker for predicting response to immune checkpoint inhibitors. Therefore, understanding its variability across patient groups, particularly between sexes, and its underlying biological determinants is of critical importance.

**Methods:**

We analyzed autosomal TMB and its association with DNA repair–related regulatory mechanisms in gastric cancer across TCGA-STAD and independent targeted sequencing cohorts. Sex-stratified analyses were integrated with gene expression, promoter methylation, and regression modeling.

**Results:**

Female tumors exhibited significantly higher autosomal TMB compared with male tumors, with differences most pronounced in older female patients. Across tumors, TMB was strongly associated with reduced MLH1 expression and increased MLH1 promoter methylation, while female tumors exhibited significantly higher MLH1 methylation levels. These relationships are consistent with mismatch repair deficiency as a major driver of mutation accumulation. In multivariable regression models adjusting for MLH1 methylation and expression, the association between sex and TMB was attenuated, suggesting that MLH1-related processes contribute to the observed sex differences. Subtype-aware analyses further suggested that the female-associated TMB elevation was partly related to increased representation of MSI/MMR-deficient tumors among females, while female and male MSI tumors showed comparable TMB. Independent targeted sequencing cohorts showed consistent directional trends, although these did not reach statistical significance.

**Conclusions:**

Together, our findings indicate that sex differences in mutation burden are linked to MLH1-associated epigenetic regulation, mismatch repair deficiency, and MSI/MMR-deficient tumor biology. These results highlight the importance of incorporating sex-specific molecular context into the interpretation of genomic biomarkers and support a more refined, biologically informed approach to precision oncology.

**Supplementary Information:**

The online version contains supplementary material available at 10.1186/s12885-026-16327-1.

## Introduction

Gastrointestinal cancers account for approximately one quarter of global cancer incidence and nearly one third of cancer-related mortality, with substantial geographical, socioeconomic, age, and sex disparities observed across gastrointestinal malignancies [1, 2]. Among these malignancies, gastric cancer (GC) remains one of the most common and lethal cancers, ranking fifth in incidence and fourth in cancer-related mortality, accounting for approximately 7.7% of all cancer deaths [3, 4]. Notably, GC incidence and mortality rates are consistently two- to three-fold higher in men than in women across diverse populations, with marked variation between regions such as East Asia, Europe, and North America [3, 5].

Tumor mutational burden (TMB) has emerged as an important biomarker for predicting response to immune checkpoint inhibitors (ICIs) and has been approved as a companion diagnostic for pembrolizumab therapy [6, 7]. Consequently, sex-associated differences in immunotherapy outcomes and their relationship with TMB have been intensively studied. However, reports remain inconsistent across cancer types and cohorts. While several pan-cancer studies [8–10] and analyses of bladder cancer [11] have reported higher TMB and improved immunotherapy responses in males, other studies have demonstrated higher TMB and superior outcomes in females with lung cancer [12], and meta-analyses [13] have failed to detect a consistent sex effect across advanced malignancies.

Importantly, inconsistencies extend beyond the predictive value of TMB for immunotherapy outcomes to baseline TMB differences between sexes, which also vary across studies. Specifically, while numerous studies have reported higher TMB values in male cancers [8, 14–16], others have found higher TMB values in females [17], and some have observed no significant differences between the sexes [18, 19]. Additionally, differences in TMB values have been shown to correlate with age [17] and ethnicity [19]. These discrepancies are further compounded by methodological heterogeneity across studies, including differences in sequencing platforms, gene panel size, and whether TMB calculations include sex chromosomes or are restricted to autosomes.

While technical and methodological factors can obscure the interpretation of sex-associated genomic differences, accumulating evidence supports sex as an important biological variable shaping the genomic landscape of non-reproductive cancers, underscoring the need for careful, context-aware evaluation of sex-related genomic differences. Recent pan-cancer analyses have revealed that molecular sex disparities are pervasive across the genome, manifesting in distinct patterns of somatic mutations, copy-number alterations, epigenetic regulation, and gene regulatory network architecture [20]. In addition to sex-chromosome effects, autosomal driver mutations also exhibit sex-biased frequencies across cancers, underscoring that these differences arise from both chromosomal dosage and autosomal mutational processes. For example, mutations in TP53 and CDKN2A are often more prevalent in males, while EGFR and PIK3CA alterations show enrichment in female cohorts across various solid tumors [16]. These genes exemplify how sex bias can appear in driver identity and pathway engagement, not just mutation counts. Moreover, computational analyses have shown that sex-specific gene regulatory network organization can influence oncogenic pathway targeting in a tissue-dependent manner, including in gastric cancer, further supporting the need for sex-stratified genomic analyses [21].

Gastric adenocarcinoma exhibits pronounced molecular heterogeneity. The landmark TCGA classification delineates four major molecular subtypes: Epstein–Barr virus (EBV)-positive, microsatellite instability (MSI), chromosomal instability (CIN), and genomically stable (GS), which differ in their underlying mutational processes, structural variation, and patterns of genomic instability [22, 23]. This diversity provides an important biological context for interpreting sex-associated genomic differences and underscores the need for integrative analyses that examine sex effects across multiple genomic layers rather than within a single molecular dimension.

In gastric cancer, elevated TMB may arise from mismatch repair deficiency (MMRD), pathogenic POLE mutations, or other endogenous and exogenous mutagenic processes, each with distinct biological and clinical implications [18]. These mechanisms reflect diverse sources of genomic instability that are not fully captured by mutation counts alone.

Among these, mismatch repair deficiency, most commonly driven by epigenetic silencing of MLH1, represents a central mechanism underlying microsatellite instability and hypermutation [22, 24, 25]. MLH1 promoter methylation is strongly associated with MSI and elevated mutational burden, yet has not been consistently linked to patient sex across studies, leaving its potential role in sex-associated mutational differences unresolved. Beyond transcriptional regulation, sex-associated genomic disparities may partly arise from differential DNA repair efficiency, a process that fundamentally shapes mutational landscapes and is biologically modulated by both age and sex.

Defects in DNA damage response and repair pathways contribute to genomic instability and can create therapeutically actionable vulnerabilities in cancer cells [26–29]. To evaluate whether sex-associated differences in gastric cancer mutation burden were accompanied by altered expression of selected repair pathways, we examined thirteen DNA-repair genes representing core mismatch repair (MLH1, MSH2, MSH6, PMS2), homologous recombination and double-strand break or replication-stress response (ATM, ATR, BRCA1, BRCA2, RAD51, RAD51C, RAD51D), and additional repair-related mechanisms including nucleotide excision/cross-link repair and alternative end-joining (ERCC1, POLQ) [26, 27].

These findings suggest that distinct molecular profiles observed in male and female patients may reflect sex-contingent variation in DNA maintenance and repair fidelity across the lifespan [30, 31]. More broadly, they highlight that sex-associated cancer disparities are likely to manifest across multiple genomic layers, including mutational processes, structural variation, and DNA repair capacity, rather than being solely reflected in steady-state transcriptional differences or pathway activation. Gastric cancer, characterized by pronounced sex bias, marked molecular heterogeneity, and diverse mutational etiologies, therefore represents a compelling model for dissecting sex-specific genomic architecture using a multi-omics approach.

In this study, we perform a comprehensive, sex-stratified analysis of gastric cancer using the TCGA-STAD cohort, focusing on the relationship between tumor mutational burden, DNA repair gene expression, and epigenetic regulation. We specifically investigate whether MLH1-associated mismatch repair deficiency contributes to sex-associated differences in mutational burden and evaluate the consistency of these patterns across independent cohorts. Through this approach, we aim to clarify the molecular basis of sex disparities in gastric cancer.

## Methods

This study integrates somatic mutation, transcriptomic, and epigenetic pattern analyses to investigate tumor-intrinsic sex differences in gastric cancer. Unless otherwise specified, all analyses were restricted to autosomal genes to avoid trivial confounding effects driven by sex chromosome dosage.

### Data sources and cohort assembly

Somatic mutation and gene expression data were obtained from The Cancer Genome Atlas (TCGA) via the Genomic Data Commons (GDC) portal, stomach adenocarcinoma gastric cancer (STAD) [32].

Somatic mutation data for TCGA-STAD, FM-AD, Genie-MSK and Genie-DFCI was analyzed using Mutation Annotation Format (MAF) files. For MSK-EGC Esophageal/Stomach cancer cohort somatic mutation data was retrieved from the processed genomic variation egc_mskcc_2020.tar.gz file obtained from cBioPortal database.

Targeted sequencing data for gastric cancer were obtained from the Foundation Medicine Adult Cancer dataset (FM‑AD) [18] the AACR Project GENIE consortium, the MSK and DFCI contributing centers [33] and the Esophageal/Stomach cancer cohort MSK-EGC 2020 (at cBioPortal.org) [34].

Samples were excluded if biological sex was missing or annotated as “other.” Final sample sizes were: TCGA‑STAD (283 male, 158 female), FM‑AD (266 male, 220 female), AACR Project GENIE-MSK (122 male, 89 female), AACR Project GENIE-DFCI (92 males, 58 female) and MSK-EGC Esophageal/Stomach cancer cohort (418 male, 69 female).

Molecular subtype annotations for TCGA stomach adenocarcinoma (TCGA-STAD) tumors were obtained from TCGA PanCancer subtype annotations. In the 441 tumors included in the primary TMB cohort, PanCancer subtype annotations identified chromosomal instability (CIN; *n* = 223), MSI/hypermutated-indel (*n* = 73), genomically stable (GS; *n* = 50), EBV-associated (*n* = 30), and POLE-like/hypermutated-SNV (*n* = 7) tumors; 58 tumors were unclassified or lacked matched subtype annotation.

MSI status was obtained from the UCSC Xena Browser TCGA-STAD clinical matrix field CDE_ID_3226963, corresponding to the TCGA-STAD MSI annotation. MSI-H tumors were classified as MSI, whereas MSS and MSI-L tumors were classified as non-MSI for binary MSI-group analyses.

These subtypes are known to differ substantially in tumor mutation burden, dominant mutational processes, and patterns of copy-number alteration [22, 23, 35]. Given the limited sample sizes within individual subtypes, particularly after sex stratification, primary analyses were conducted at the pan-STAD level to maximize statistical power for detecting global sex-associated genomic patterns. Subtype-aware analyses were subsequently performed to evaluate whether the observed TMB patterns were related to molecular subtype composition.

Sample numbers differed slightly across analyses because mutation-burden, RNA-seq expression, promoter methylation, MSI annotation, and molecular subtype data were available for partially overlapping TCGA-STAD tumor subsets. Each analysis was therefore performed using the largest complete-case subset available for the required data types.

### Autosomal restriction strategy

To focus on tumor-intrinsic sex differences and avoid trivial effects arising from sex-chromosome dosage, all genome- and transcriptome-based analyses were restricted to autosomal genes. Genes located on chromosomes X, Y, and mitochondrial DNA (chrM) were excluded. Only genes mapped to chromosomes 1–22 were retained for all subsequent analyses.

### Somatic mutation processing and tumor mutational burden

#### Somatic mutation data

Only non-silent, coding somatic mutations in autosomal genes were considered for mutation-burden analyses, including missense, nonsense, frameshift, and splice-site variants.

#### Tumor mutational burden (TMB)

Tumor mutational burden was calculated separately for each sample as the number of non-silent somatic mutations in autosomal coding regions divided by the size of the interrogated autosomal coding region (in megabases). The primary sex-stratified TMB analysis included 441 TCGA-STAD tumors with available mutation-burden and sex annotation. Only single-nucleotide variants and small insertions/deletions were included. For TCGA exome data, mutation counts were normalized by 35.8 megabases (Mb), representing the estimated size of the autosomal exome. For targeted panels, normalization was performed using panel-specific autosomal coding sizes: 0.78 Mb for FM‑AD (309 genes), 1.15 Mb for GENIE‑MSK (472 genes), and 1.12 Mb for GENIE-DFCI (~ 450 genes), and inferred panel size of 0.90 to 1.16 Mb for MSK-EGC cohort (341–468 genes, respectively).

#### Tumor variation burden sensitivity analysis in targeted sequencing cohorts

To evaluate whether the external validation results were affected by panel-size normalization or by the strict nonsynonymous TMB definition, we performed tumor variation burden sensitivity analyses in the independent targeted-sequencing cohorts. First, we compared raw autosomal nonsynonymous mutation counts per tumor, corresponding to the numerator of the recalculated TMB metric before division by panel size. Second, we inspected the full set of autosomal somatic variant classifications available in each targeted-panel mutation file and calculated the number of additional captured variants beyond the nonsynonymous classes used for TMB estimation. Sex-stratified comparisons were performed using Welch’s t-test.

### Statistical framework

All analyses were conducted using Python (v3.12), SciPy (v1.16.3), and R (v4.4.2). Python was used for data processing and statistical testing, while R was used for normalization, pathway analyses, and visualization. Unless otherwise specified, all statistical tests were two-sided. Group comparisons were performed using Mann–Whitney U, Kruskal–Wallis, or Welch’s t-test as appropriate. Associations between continuous variables were assessed using Spearman rank correlations calculated with the scipy.stats.spearmanr function in Python/SciPy. Spearman’s rank correlation coefficient was defined as the Pearson correlation between the ranked variables: ρ = cov(R(x), R(y)) / [σR(x)σR(y)], where R(x) and R(y) are the ranks of the paired observations [36]. Categorical comparisons were evaluated using Fisher’s exact test or chi-square tests as indicated. Linear regression models were used to assess interaction effects. Multiple hypothesis testing was controlled using the Benjamini–Hochberg procedure, with FDR < 0.05 considered statistically significant unless otherwise specified.

### DNA-repair gene expression and mutational correlates

Expression levels of thirteen canonical DNA-repair genes were correlated with tumor mutation burden using tumors with available RNA-seq expression and TMB data. The selected genes were chosen to represent major DNA-repair pathways with established relevance to genomic instability and cancer therapy, including mismatch repair, homologous recombination and replication-stress signaling, nucleotide excision/cross-link repair, and alternative end-joining. For DNA-repair gene analyses, Spearman rank correlations were computed in the combined cohort and separately within female and male tumors, using samples with non-missing values for each gene-TMB pair. Analyses required at least 10 evaluable samples per comparison; no tests were excluded under this criterion. Multiple testing correction was applied using BH-FDR across all gene-TMB comparisons. Associations with BH-FDR < 0.05 were considered statistically significant. For ATM- and MLH1-focused analyses integrating promoter methylation, gene expression, and TMB, analyses were restricted to the corresponding complete-case subsets. MLH1 analyses incorporating MSI annotation were restricted to the complete-case overlap, which included 373 TCGA-STAD tumors.

### Gene methylation level analysis

DNA methylation profile analysis was performed utilizing TCGA Illumina Infinium HumanMethylation450 BeadChip data.

Promoter probes of the MLH1 and ATM genes were retrieved from the UCSC table browser (assembly GRCh37/hg19; Illumina 450 K “snpArrayIllumina” table). Promoter-associated probes were selected based on genomic proximity to transcription start sites and prior annotation in Illumina 450 K arrays. MLH1 probes that were analyzed included: cg23658326, cg11600697, cg21490561 and cg00893636; ATM probes that were analyzed included cg01756564, cg22477592, cg05231509, cg24027342, cg03092399, cg19643901, cg19892525, cg12139707, cg05471373, cg27627854, cg04770025, cg02511767, cg17066171, cg18474718, cg08954307, cg15535686, and cg21681962.

## Results

### Sex-associated differences in autosomal TMB in TCGA stomach adenocarcinoma cohort

Autosomal tumor mutational burden (TMB) was evaluated in 441 TCGA-STAD tumors with available mutation-burden and sex annotation, including 158 female and 283 male tumors.

**Fig. 1:**
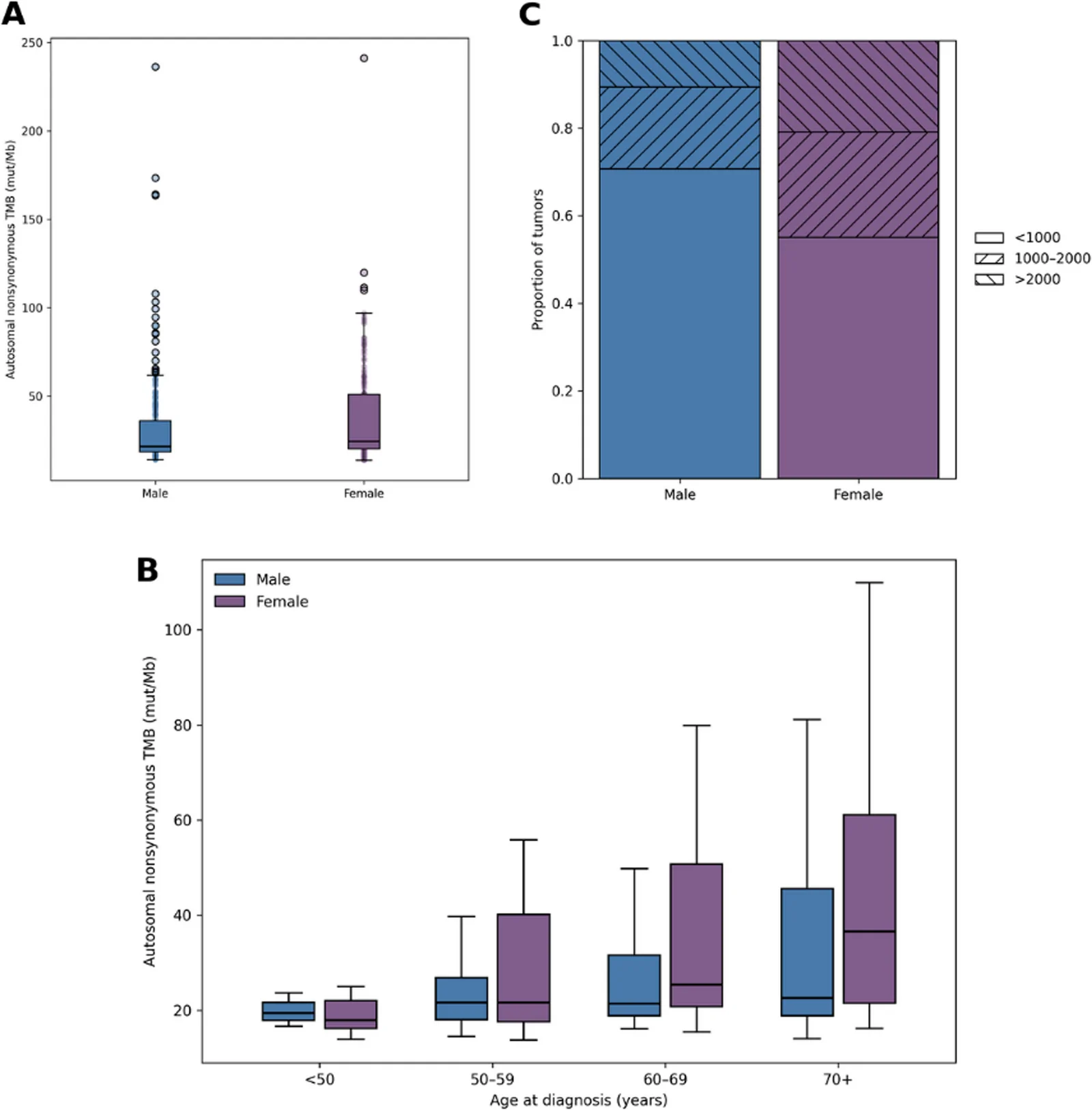
Sex differences in tumor mutational burden and its age-dependent distribution in gastric cancer. **A** Distribution of autosomal nonsynonymous tumor mutational burden (TMB) in male and female TCGA-STAD tumors. Female tumors exhibited higher TMB than male tumors (Mann–Whitney U test, p = 6.82 × 10⁻⁴). **B** TMB stratified by age at diagnosis. Within-age-group sex comparisons were evaluated using Mann–Whitney U tests: <50 years, p = 0.259; 50–59 years, p = 0.750; 60–69 years, p = 0.036; and ≥70 years, p = 0.00277. **C** Distribution of tumors across TMB categories (<1000, 1000–2000, and >2000 mutations). TMB-category distribution differed by sex (chi-square test, p = 1.75 × 10⁻³), with female tumors enriched in the >2000-mutation category (Fisher’s exact test, p = 4.31 × 10⁻³)

A significant sex difference in autosomal TMB was observed with higher TMB values in female tumors compared with male tumors (40.5 mut/Mb versus 32.3 mut/Mb, *p* = 0.00032; Fig. 1A).

Autosomal TMB increased with age in both sexes. Age-stratified analyses within TCGA-STAD showed that the female-biased TMB difference was most pronounced in the 61–70 and 71–80 age groups, where mean TMB values were higher in females than in males (< 50 17.8 mut/Mb vs. 19.4 mut/Mb, *P* = 0.26; 50–59 21.59 mut/Mb vs. 21.57 *p* = 0.75; 60–69 25.33 mut/Mb vs. 21.39 *p* = 0.036; >70 36.51 vs. 22.57 *p* = 0.0028; Fig. 1B). These findings indicate that the elevated mutation burden observed in female gastric cancers is primarily driven by tumors diagnosed at older ages.

Stratification of tumors by mutation burden revealed enrichment of hypermutated tumors in female samples. Tumors were classified according to three mutation burden categories: <1000, 1000–2000 and > 2000 mutations per sample. Female gastric cancers exhibited higher TMB primarily due to enrichment of hypermutated tumors (Fig. 1C).

### DNA repair gene expression associates with TCGA-STAD mutational burden

To investigate whether transcriptional variation in DNA repair genes is associated with gastric cancer mutation burden, expression levels of thirteen canonical DNA repair genes were correlated with autosomal tumor mutational burden (TMB) in the TCGA stomach adenocarcinoma cohort. Analyses were restricted to tumors with complete RNA-seq expression and TMB data, yielding 411 TCGA-STAD samples. Associations were evaluated using Spearman correlations, with Benjamini–Hochberg correction applied for multiple testing across all genes.

Eight of the thirteen genes exhibited significant correlations with TMB (RAD51, MLH1, MSH2, ATM, POLQ, RAD51C, BRCA1 and BRCA2; all BH-FDR < 0.05; Table S1). Among these genes, MLH1 exhibited the strongest inverse association with mutation burden (Spearman ρ ≈ −0.29, BH-FDR = 1.4 × 10⁻⁸), indicating that tumors with lower MLH1 expression accumulated substantially higher numbers of somatic mutations. ATM expression also showed a moderate inverse association with TMB (Spearman ρ ≈ −0.24, BH-FDR < 4.1 × 10⁻⁶).

In contrast, several homologous recombination repair (HRR) genes, including RAD51, RAD51C, POLQ and BRCA1, as well as the mismatch repair gene MSH2, exhibited positive correlations with TMB (Table S1). Tumors with higher expression of these genes tended to have elevated mutation burdens, consistent with a reactive DNA damage response rather than a primary mutational driver. These patterns suggest that classical homologous recombination deficiency–driven mutational processes are unlikely to represent the dominant mechanism shaping mutational burden in the TCGA-STAD cohort.

To further evaluate these associations, tumors were stratified into three mutational burden groups (< 1000, 1000–2000, and > 2000 mutations). MLH1 expression showed the strongest stratified association, with markedly reduced expression in hypermutated tumors (Kruskal–Wallis FDR = 1.6 × 10⁻¹⁵). In contrast, other DNA repair genes demonstrated modest increases in expression with increasing mutation burden, consistent with activation of DNA repair pathways in response to elevated genomic damage rather than primary drivers of hypermutation (Table S2).

These observations prompted further investigation of whether promoter methylation of the DNA repair genes inversely associated with mutational burden might contribute to the observed expression–TMB relationship.

### Sex-specific MLH1 methylation and expression are associated with microsatellite instability and hypermutation in TCGA-STAD tumors

#### Inverse associations of DNA repair genes with mutational burden highlight MLH1 as a candidate regulator of mutation burden

Among DNA repair genes, MLH1 showed a strong inverse association with mutational burden in TCGA-STAD tumors, whereas ATM exhibited a more modest effect (Kruskal–Wallis test, BH-FDR = 1.62 × 10⁻¹⁵ and 3.31 × 10⁻³, respectively). Given the central role of MLH1 in mismatch repair and ATM in DNA damage response and cell-cycle checkpoint regulation, we examined whether promoter methylation of these genes contributes to the observed expression–mutation burden relationships by evaluating associations among promoter methylation, gene expression, mutational burden, microsatellite instability (MSI), and sample sex in TCGA-STAD tumors.

#### Promoter methylation of DNA repair genes reveals gene-specific associations with mutational burden

Promoter methylation of ATM exhibited a narrow distribution across tumors and showed no association with gene expression, mutation burden, or sample sex (Fig. S1), suggesting that ATM promoter methylation is unlikely to represent a regulatory mechanism contributing to mutational burden variation in TCGA-STAD tumors.

In contrast, MLH1 promoter methylation was strongly associated with MLH1 expression, mutational burden, and MSI status, and also differed by sex across TCGA-STAD samples, highlighting MLH1 as a key epigenetically regulated driver of the hypermutated phenotype observed in gastric tumors.

**Fig. 2:**
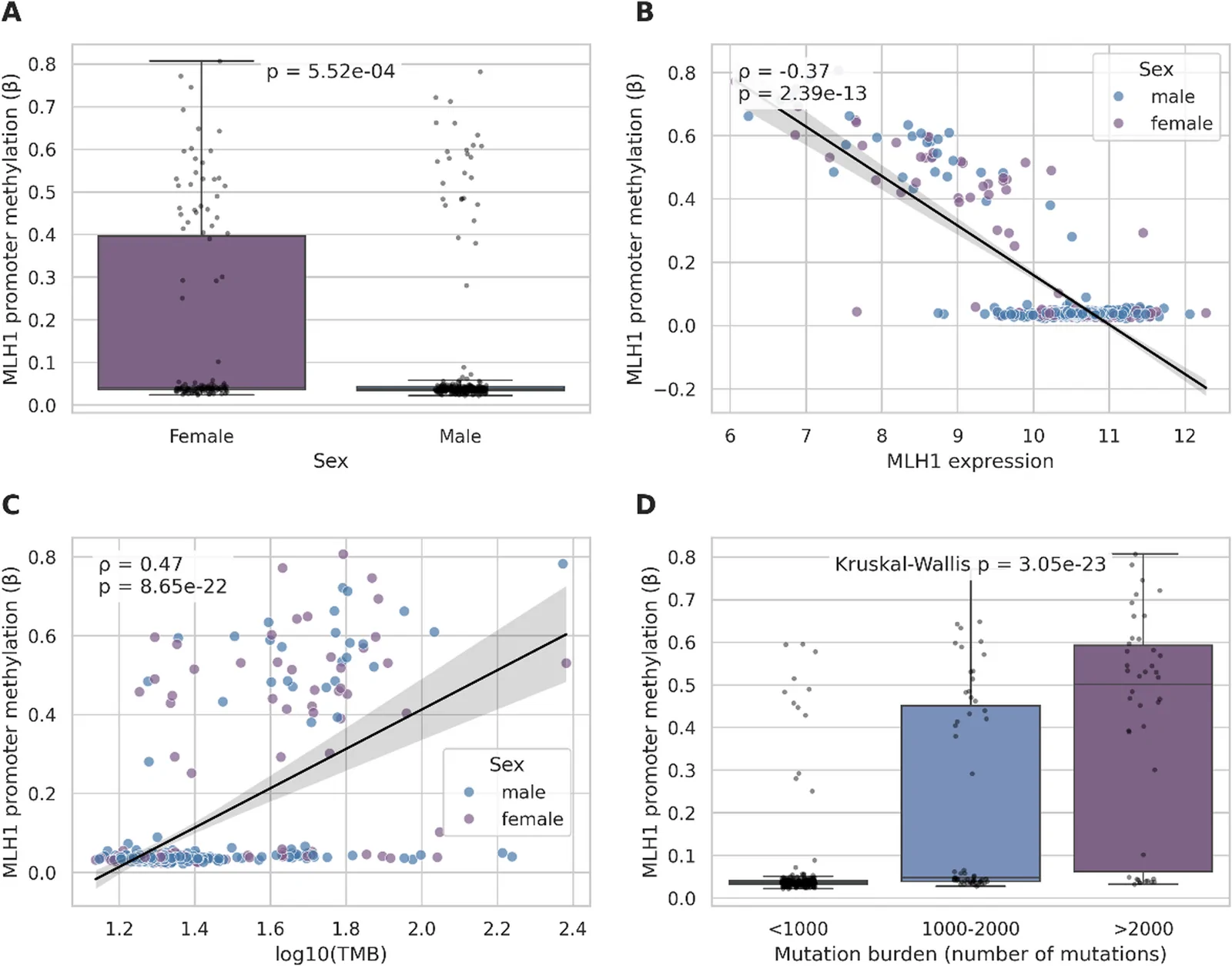
MLH1 promoter methylation is associated with MLH1 expression and mutation burden in TCGA-STAD tumors. **A** MLH1 promoter methylation levels stratified by sex. Female tumors showed modestly higher methylation levels than male tumors (median β = 0.0408 vs 0.0371; Mann–Whitney p = 5.52 × 10⁻⁴). **B** Relationship between MLH1 promoter methylation and MLH1 expression across tumors (n = 412). MLH1 promoter methylation was significantly inversely correlated with MLH1 expression (Spearman ρ = −0.37, p = 2.39 × 10⁻13), consistent with epigenetic silencing. **C** Association between MLH1 promoter methylation and autosomal tumor mutational burden. MLH1 methylation showed a strong positive correlation with log10(TMB) (Spearman ρ = 0.47, p = 8.65 × 10⁻²²). **D** MLH1 promoter methylation stratified by mutation burden categories (<1000, 1000–2000, and >2000 mutations). Tumors with >2000 mutations exhibited substantially elevated MLH1 methylation (median β ≈ 0.50) compared with tumors with lower mutation burdens (median β ≈ 0.036–0.049) (Kruskal–Wallis p = 3.05 × 10⁻²³), consistent with mismatch repair deficiency associated with MLH1 promoter hypermethylation

MLH1 promoter methylation differed significantly between sexes, with female tumors showing modestly higher methylation levels than male tumors (median β = 0.0408 vs. 0.0371; Mann–Whitney *p* = 5.52 × 10⁻⁴; Fig. 2A).

Across tumors, MLH1 promoter methylation was inversely correlated with MLH1 expression (Spearman ρ = −0.37, *p* = 2.39 × 10⁻¹³; Fig. 2B), consistent with epigenetic silencing of MLH1 through promoter hypermethylation. Tumors clustered near β ≈ 0 represent cases with little or no detectable MLH1 promoter methylation at the assayed CpG sites; these low-methylation tumors span a range of MLH1 expression and TMB values, indicating that promoter methylation is not the sole determinant of these phenotypes and that additional regulatory or mutational mechanisms may also contribute. MLH1 promoter methylation was also positively associated with autosomal tumor mutational burden (Spearman ρ = 0.47, *p* = 8.65 × 10⁻²²; Fig. 2C), with elevated methylation most pronounced in the high-mutation-burden subset.

When tumors were stratified by mutation burden (< 1000, 1000–2000, and > 2000 mutations), MLH1 methylation increased markedly with mutation burden (Kruskal–Wallis *p* = 3.05 × 10⁻²³). Tumors with > 2000 mutations exhibited substantially elevated methylation (median β ≈ 0.502) compared with tumors with lower mutation burdens (median β 0.036–0.049), consistent with mismatch repair deficiency associated with MLH1 promoter silencing (Fig. 2D).

Hypermutated tumors (> 2000 mutations) were more frequent among female samples (21/141, 14.9%) than male samples (25/271, 9.2%), consistent with the observed association between MLH1 methylation and hypermutation, although this difference did not reach statistical significance (odds ratio = 1.72; Fisher’s exact test *p* = 0.099; Fig. S2).

Consistent with these observations, MSI tumors exhibited markedly higher MLH1 promoter methylation and reduced MLH1 expression compared with non-MSI tumors, alongside substantially elevated mutation burden (Fig. S3). In addition, analysis of MLH1 methylation distribution revealed a transition toward hypermutated tumors at intermediate-to-high methylation levels (β ≈ 0.35), consistent with a threshold-like relationship between MLH1 promoter methylation and the emergence of the hypermutated phenotype (Fig. S4).

Notably, MSI tumors were diagnosed at significantly older ages compared with non-MSI tumors (Fig. S5; Mann–Whitney *p* = 4.69 × 10⁻³). This age shift is consistent with the enrichment of hypermutated tumors in older patients and supports the inclusion of age as a relevant covariate in analyses of MSI-associated mutational processes.

#### MLH1 expression is linked to MSI and hypermutation in a sex-stratified context

Building on the observed association between MLH1 promoter methylation, gene expression, and mutational burden, we next examined whether MLH1 expression is linked to microsatellite instability (MSI) and hypermutation in a sex-stratified context.

**Fig. 3:**
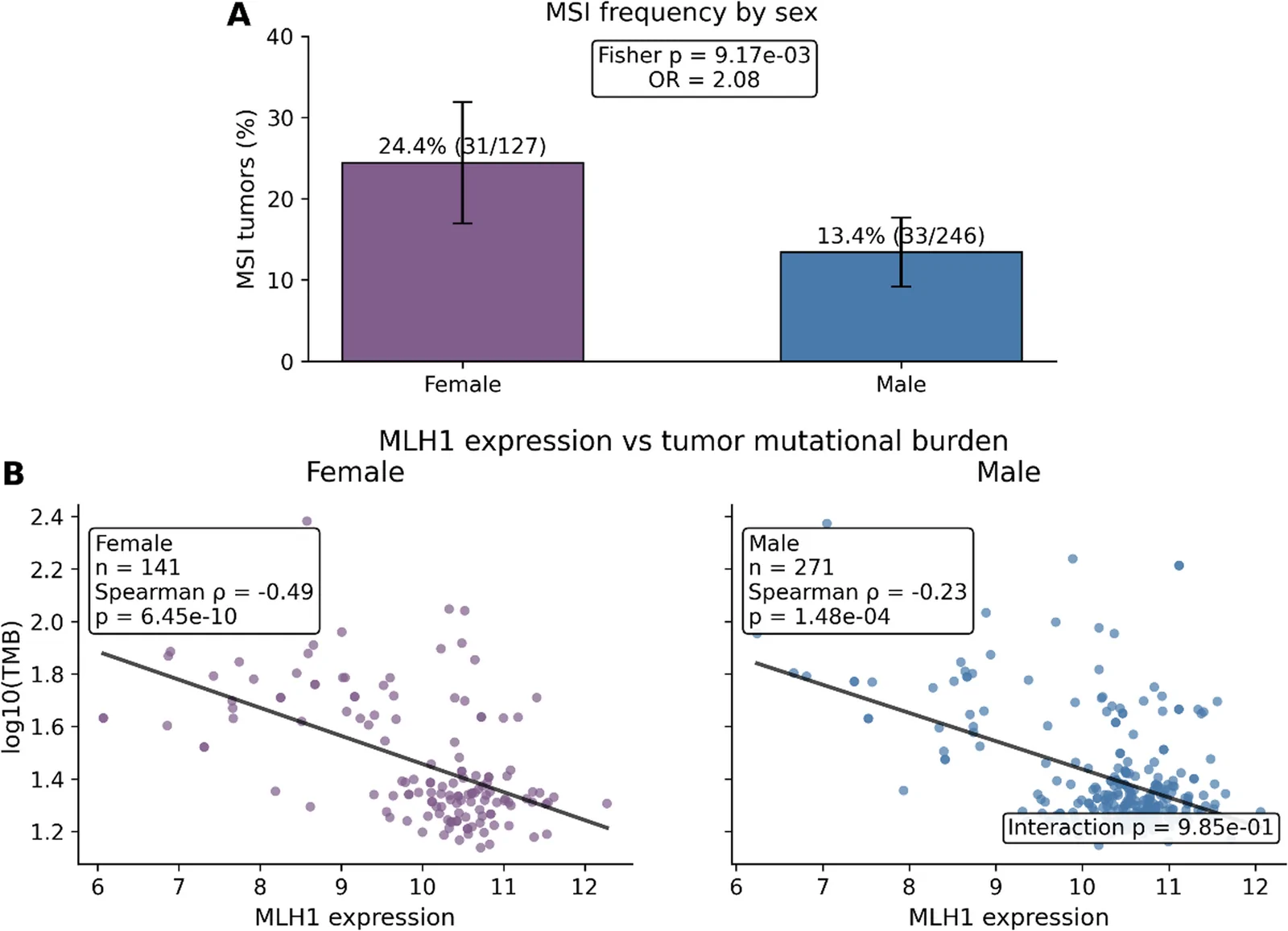
MLH1 expression and MSI-associated hypermutation in TCGA-STAD tumors. **A** Frequency of microsatellite instability (MSI) tumors stratified by sex. MSI tumors were significantly more frequent in females (31/127, 24.4%) than in males (33/246, 13.4%) (Fisher’s exact test p = 9.17 × 10⁻³; odds ratio = 2.08). Error bars represent 95% confidence intervals for the estimated proportions. **B** Relationship between MLH1 expression and tumor mutational burden (TMB) within sex. Scatterplots show MLH1 expression versus log10-transformed autosomal TMB for female (left) and male (right) tumors. MLH1 expression was negatively correlated with mutation burden in both females (Spearman ρ = −0.49, p = 6.45 × 10⁻¹⁰, n = 141) and males (Spearman ρ = −0.23, p = 1.48 × 10⁻⁴, n = 271). Linear regression lines are shown for visualization. A linear regression model of log10(TMB) including MLH1 expression, sex, and the MLH1 expression × sex interaction showed no significant interaction effect (βinteraction = 0.00034, p = 0.985)

MSI tumors were characterized by markedly reduced MLH1 expression and substantially elevated mutation burden compared with non-MSI tumors, consistent with mismatch repair deficiency as a driver of the hypermutated phenotype (Fig. 3A).

Within both sexes, MLH1 expression was significantly inversely associated with log10(TMB), as assessed by linear regression (female: *p* = 6.45 × 10⁻¹⁰; male: *p* = 1.48 × 10⁻⁴; Fig. 3B). Although the inverse association between MLH1 expression and mutational burden appeared more pronounced in female tumors, formal testing did not support a statistically significant sex-dependent difference (interaction β ≈ 0.00034, *p* = 0.985).

### Subtype-aware tumor mutational burden analyses in TCGA-STAD

To evaluate whether the observed sex-associated TMB difference was related to molecular subtype composition, we performed subtype-aware sensitivity analyses using TCGA PanCancer subtype annotations. Among the 441 tumors in the primary TMB cohort, 223 were classified as chromosomal instability (CIN), 73 as MSI/hypermutated-indel, 50 as genomically stable (GS), 30 as EBV-associated, 7 as POLE-like/hypermutated-SNV, and 58 were unclassified or lacked matched subtype annotation (Table S6). Consistent with the MSI analysis shown in Fig. 3A, MSI tumors were more frequent among females than males. In the primary TMB cohort, 38/158 female tumors and 35/283 male tumors were classified as MSI/hypermutated-indel by PanCancer subtype annotation. Within individual molecular subtypes, TMB did not differ significantly between female and male tumors, including CIN (*p* = 0.058), MSI (*p* = 0.703), GS (*p* = 0.161), and unclassified tumors (*p* = 0.975; Fig. S6A). In contrast, MSI tumors showed substantially higher TMB than non-MSI tumors overall (*p* = 6.78 × 10⁻³²) and within both female (*p* = 2.70 × 10⁻¹⁴) and male tumors (*p* = 1.91 × 10⁻¹⁷; Fig. S6B). Together, these subtype-aware analyses suggest that the female-associated TMB elevation was partly related to increased representation of MSI/MMR-deficient tumors among females, while female and male MSI tumors showed comparable TMB.

### External validation cohorts

To assess whether the observed sex differences in autosomal TMB were reproducible in independent datasets, we examined three targeted sequencing cohorts (Table S3).

In the FM-AD cohort (220 females, 266 males), mean autosomal TMB was higher in female tumors than in male tumors (9.96 ± 12.43 vs. 9.29 ± 10.73 mutations/Mb), consistent with the direction observed in TCGA-STAD, although this difference was not statistically significant (Welch’s t-test, *p* = 0.53).

In the AACR Project GENIE gastric cancer cohort, autosomal TMB values were broadly comparable between sexes. In the GENIE-MSK subset (89 females, 122 males), mean TMB was slightly higher in male tumors (5.62 ± 8.49 mutations/Mb) than in female tumors (4.32 ± 9.75 mutations/Mb), whereas in the GENIE-DFCI subset (58 females, 92 males), mean TMB was marginally higher in female tumors (8.21 ± 9.42 mutations/Mb) than in male tumors (8.03 ± 8.93 mutations/Mb); neither comparison reached statistical significance (Welch’s t-test, *p* = 0.32 and *p* = 0.91, respectively).

We further evaluated an independent MSK-EGC cohort (69 females, 418 males) sequenced using MSK-IMPACT panels. Mean autosomal TMB was higher in female tumors (7.41 ± 8.94 mutations/Mb) than in male tumors (5.98 ± 7.68 mutations/Mb), although this difference did not reach statistical significance (Welch’s t-test, *p* = 0.21).

Raw autosomal nonsynonymous mutation counts yielded results similar to the normalized TMB analysis, as expected because these counts represent the numerator of the recalculated panel TMB metric. Inspection of all available autosomal somatic variant classifications showed that the targeted-panel cohorts were dominated by nonsynonymous coding mutations. Additional synonymous, splice-region, intronic, untranslated-region, and flanking variants were negligible in FM-AD, GENIE-MSK, and MSK-EGC, representing 1.3%, 0.2%, and 0.2% of the nonsynonymous mutation count, respectively. GENIE-DFCI contained the largest additional captured-variant component, with 136 additional variants beyond 1,358 nonsynonymous mutations, representing approximately 10% of the nonsynonymous count. Sex-stratified expanded TVB analysis in GENIE-DFCI showed no material change compared with raw nonsynonymous TVB. Variant-class composition and sex-stratified TVB comparisons are provided in Tables S4 and S5.

Across these targeted sequencing cohorts, mean autosomal TMB was higher in female tumors in three of four comparisons; however, none of these differences reached statistical significance. This lack of consistent significance likely reflects the limited genomic footprint of targeted panels, which limits the accurate estimation of genome-wide mutational burden.

## Discussion

Cancer incidence, progression, and treatment response differ between men and women, contributing to persistent sex disparities in cancer outcomes [37–39]. These differences highlight the importance of incorporating sex as a biological variable in cancer research and genomic interpretation.

In this study, we identify sex-associated differences in the somatic mutational landscape of gastric cancer, primarily manifested at the level of tumor mutational burden (TMB) and its relationship with DNA repair–related processes. Specifically, we observe higher autosomal TMB in female gastric tumors compared with male tumors in the TCGA-STAD cohort, indicating that sex-associated differences in mutation accumulation represent a key dimension of gastric cancer genomic heterogeneity. Notably, this difference was driven by higher TMB in older female patients, indicating that mutation accumulation in gastric cancer may be modulated by sex-specific, age-related factors.

Given the widespread use of TMB as a biomarker for immunotherapy eligibility, these findings raise the possibility that baseline sex differences in mutation burden may influence threshold-based treatment decisions in gastric cancer. While prospective validation is required, uniform TMB cutoffs may not fully capture sex-dependent genomic contexts. These findings align with recent reports suggesting that sex may influence the interpretation of TMB as a biomarker for immunotherapy [10, 13, 40].

Across independent targeted sequencing cohorts, including the Foundation Medicine Adult Cancer dataset (FM-AD), AACR Project GENIE (MSK and DFCI subsets), and the MSK-EGC cohort, three of four datasets demonstrated a consistent directional trend toward higher TMB in female tumors. While statistical significance was not reached, this consistency across cohorts supports the robustness of the observation despite differences in sequencing platforms and cohort composition. The lack of statistical significance is likely attributable to the limited genomic footprint of targeted panels, which interrogate only a small fraction of the exome and thereby reduce the resolution and dynamic range of TMB estimation. These findings suggest that limited genomic coverage may attenuate the detection of biologically meaningful sex-associated differences observable in whole-exome datasets.

The findings presented here raise a central biological question concerning the mechanisms underlying sex-associated differences in mutation burden. Our results point to DNA repair–related processes, particularly mismatch repair deficiency, as a key contributing factor. Among DNA repair genes examined, MLH1 exhibited the strongest inverse association with mutation burden, consistent with its established role in maintaining genomic integrity through mismatch repair. In contrast, positive associations observed for several homologous recombination–related genes likely reflect compensatory responses to increased genomic instability rather than primary drivers of mutation accumulation.

MLH1 promoter methylation emerged as a central feature linking epigenetic regulation, mismatch repair deficiency, and mutation burden. Consistent with prior studies, MLH1 methylation was strongly associated with reduced MLH1 expression, microsatellite instability (MSI), and elevated mutational burden. Extensive literature has established that epigenetic silencing of MLH1 is a principal mechanism underlying MSI and hypermutation in gastric cancer [24, 25, 41]. In addition, altered expression of mismatch repair genes, including MLH1 and MSH2, has been reported in gastric tumors and linked to MSI-associated genomic instability [42], further supporting the role of mismatch repair disruption in shaping mutation accumulation.

To date, MLH1 promoter methylation has been primarily studied in the context of microsatellite instability and tumorigenesis, with limited investigation into its relationship with patient sex. In this context, our findings suggest that MLH1-associated mechanisms may contribute to sex-associated differences in mutation burden indirectly, rather than through a simple sex-specific alteration in MLH1 methylation frequency. Instead, sex may influence the broader regulatory or cellular context in which mismatch repair deficiency arises and is tolerated.

The observed higher frequency of hypermutated and MSI-high tumors among female samples further supports a mismatch repair–linked mechanism underlying sex-associated differences in mutation burden, consistent with MLH1-associated hypermutation. Given the established role of MLH1 methylation in driving MSI and hypermutation, these observations provide additional evidence that epigenetic dysregulation of mismatch repair contributes to the observed sex bias in mutation burden.

Age-stratified analyses revealed that the observed sex differences in TMB were primarily driven by higher mutation burden in older female patients, with divergence between sexes most pronounced in the post-menopausal age range. This temporal pattern suggests that sex-associated differences in genomic maintenance may evolve over time, potentially reflecting interactions between aging, hormonal changes, and DNA repair capacity. Sex hormones, particularly estrogens, have been implicated in modulating DNA repair pathways and cellular responses to genotoxic stress, and their decline with age may contribute to altered mutation accumulation dynamics [43].

Consistent with this age-dependent pattern, MSI tumors were also observed at older ages (Fig. S5), supporting a link between aging, mismatch repair deficiency, and hypermutation.

Together, these findings support a model in which sex-associated differences in gastric cancer are reflected at the level of mutational processes, DNA repair dynamics, and molecular subtype composition. Our subtype-aware analyses suggest that the female-associated TMB elevation is partly explained by higher representation of MSI/MMR-deficient tumors among females. This does not imply that female MSI tumors are more mutated than male MSI tumors; rather, sex may shape the likelihood of developing tumors with MLH1/MMR-deficient biology.

The mechanisms by which sex may influence MLH1 promoter methylation or MSI/MMR-deficient tumor biology remain uncertain. One possibility is that MLH1 methylation reflects a broader sex-biased epigenetic context. Sex differences in DNA methylation, chromatin accessibility, and gene regulation have been reported across normal and tumor tissues, including autosomal loci and DNA-repair pathways [31]. MLH1 silencing is also regulated by chromatin mechanisms beyond DNA methylation. MLH1 promoter silencing has been demonstrated in several microenvironment- or exposure-related contexts: hypoxia can induce durable MLH1 promoter silencing through loss of activating H3K4 methylation mediated by LSD1/KDM1A and PLU-1/KDM5B, whereas environmental carcinogen exposure has been linked to enrichment of the repressive H3K9me2 mark at the MLH1 promoter through the H3K9 methyltransferase G9a/EHMT2 [44, 45]. Furthermore, the MLH1 promoter-region variant rs1800734 has been associated with altered transcription-factor binding and MLH1 epigenetic regulation, and germline variation at this locus has been linked in some studies to reproductive phenotypes and female-modified lung cancer risk [46–49]. Together, these observations suggest that sex-associated differences in hormonal milieu, environmental exposure response, hypoxia, inflammatory state, or baseline chromatin configuration could influence the probability of MLH1 promoter methylation and silencing. However, our data do not establish a causal mechanism, and direct experimental studies will be required to determine whether sex modifies MLH1 epigenetic regulation in gastric cancer.

At the individual-patient level, MSI/MMR status, MLH1 methylation/expression, and TMB remain the relevant biomarkers regardless of sex. At the population level, the distribution of these biomarker-positive tumors appears sex-biased.

These findings may have clinical relevance by suggesting that MLH1 methylation and expression could provide mechanistic context for interpreting mutation burden, particularly in relation to MSI/MMR-deficient high-TMB tumors. Although TMB is increasingly used as a biomarker for immune checkpoint inhibitor response, high TMB can arise through different mutational processes, not all of which may reflect equivalent immune responsiveness. In this setting, MLH1 promoter hypermethylation and reduced MLH1 expression may help identify tumors in which elevated mutation burden is more specifically linked to mismatch-repair deficiency and MSI-associated hypermutation, a biological context associated with increased neoantigen load and clinical benefit from PD-1 blockade [50, 51]. Conversely, mismatch-repair deficiency has also been associated, particularly in colorectal cancer, with limited benefit from fluoropyrimidine-based adjuvant therapy, emphasizing that MMR status can inform treatment sensitivity beyond mutation burden alone [52]. MLH1 promoter methylation also has established diagnostic utility in clinical MMR-deficiency workups, including its use to support classification of sporadic MLH1-deficient tumors [53]. Together, these observations suggest that integrating MLH1 methylation and expression with TMB, MSI/MMR status, and sex-stratified analyses may provide a more biologically informative framework for interpreting hypermutation and immunotherapy-relevant biomarkers in gastric cancer. However, this interpretation remains hypothesis-generating, and dedicated treatment-response studies will be required to determine whether sex-stratified MLH1 regulation improves prediction of clinical benefit.

This study has several limitations. Analyses were conducted using retrospective public datasets and therefore cannot directly assess clinical outcomes or treatment responses in relation to sex-specific mutational architecture. In addition, mechanistic inferences regarding DNA repair modulation remain associative. Environmental and high-risk exposure variables were not analyzed in the present study and were not harmonized across the TCGA-STAD and targeted-panel validation cohorts, limiting our ability to evaluate whether subtype- and sex-associated TMB patterns vary by exposure context. Functional validation in experimental systems and prospective clinical cohorts will be required to establish causality and to determine the therapeutic implications of these findings. External validation was limited by the structure of the available targeted sequencing cohorts. Tumor variation burden sensitivity analyses indicated that these limitations were not primarily due to panel-size normalization, but rather to restricted genomic footprint, cancer-gene-focused panel design, and sparse or inconsistent capture of variant classes beyond nonsynonymous coding mutations. Consequently, the targeted-panel cohorts could not fully evaluate whether a broader MLH1-associated tumor variation burden recapitulates the TCGA-STAD whole-exome finding.

## Conclusions

Our study shows that sex-associated differences in gastric cancer are reflected in tumor mutation burden and are accompanied by DNA repair–related differences, particularly involving MLH1 methylation and expression. These findings support considering sex as a biological variable when interpreting tumor genomic biomarkers, including mutation burden and mismatch repair–related features. More broadly, our results highlight the potential value of sex-aware genomic frameworks in cancer research and precision oncology, while emphasizing the need for further validation, including treatment-response studies, before clinical implementation.

## Supplementary Information

Supplementary Material 1.

Supplementary Material 2.

## Data Availability

TCGA data supporting this study are publicly available through the Genomic Data Commons (GDC) portal under the TCGA-STAD project. Targeted sequencing data from the Foundation Medicine Adult Cancer (FM-AD) dataset and the AACR Project GENIE gastric cancer cohort were accessed and analyzed in accordance with their respective data use agreements. Additional targeted sequencing data from the MSK-IMPACT esophagogastric cancer cohort (MSK-EGC) were obtained from cBioPortal (https://www.cbioportal.org/) and analyzed in accordance with their data access policies. Processed data generated during this study are included in the manuscript and Supplementary Information. Additional intermediate files and analysis outputs are described in the Methods section.

## Abbreviations

AACR: American Association for Cancer Research
BH: Benjamini–Hochberg
BH-FDR: Benjamini–Hochberg false discovery rate
CI: Confidence interval
chrM: Mitochondrial chromosome
CIN: Chromosomal instability
EBV: Epstein–Barr virus
FDR: False discovery rate
FM-AD: Foundation Medicine Adult Cancer dataset
GC: Gastric cancer
GDC: Genomic Data Commons
GENIE: AACR Project GENIE
GENIE-DFCI: AACR Project GENIE Dana-Farber Cancer Institute cohort
GENIE-MSK: AACR Project GENIE Memorial Sloan Kettering Cancer Center cohort
GS: Genomically stable
HRR: Homologous recombination repair
ICI: Immune checkpoint inhibitor
MAF: Mutation Annotation Format
Mb: Megabase
MLH1: MutL homolog 1
MMR: Mismatch repair
MMRD: Mismatch repair deficiency
MSI: Microsatellite instability
MSI-H: Microsatellite instability-high
MSI-L: Microsatellite instability-low
MSK-EGC: Memorial Sloan Kettering Cancer Center Early Gastric Cancer cohort
MSS: Microsatellite stable
OR: Odds ratio
RNA-seq: RNA sequencing
SD: Standard deviation
STAD: Stomach adenocarcinoma
TCGA: The Cancer Genome Atlas
TMB: Tumor mutational burden
WES: Whole-exome sequencing
