# Supplementary material for: Sex differences in gastric cancer mutational burden reflect MLH1-associated epigenetic regulation
Source: BMC Cancer. 2026 Jun 15;26:1010. doi: 10.1186/s12885-026-16327-1 (PMC13501732; doi:10.1186/s12885-026-16327-1)
Supplement: Supplementary file 1 [file 12885_2026_16327_MOESM2_ESM.pdf]

Sex Differences in Gastric Cancer Mutational Burden Reflect MLH1-Associated Epigenetic Regulation

Noa Klein[1] and Dorit Shweiki[1*]

Corresponding author: dorits@mta.ac.il

Supplementary Materials:

- Figs. S1 to S6
- Tables S1 to S6 (attached Excel file)

Fig. S1: ATM promoter methylation shows limited variation and no consistent association with gene expression or sex in TCGA-STAD tumors

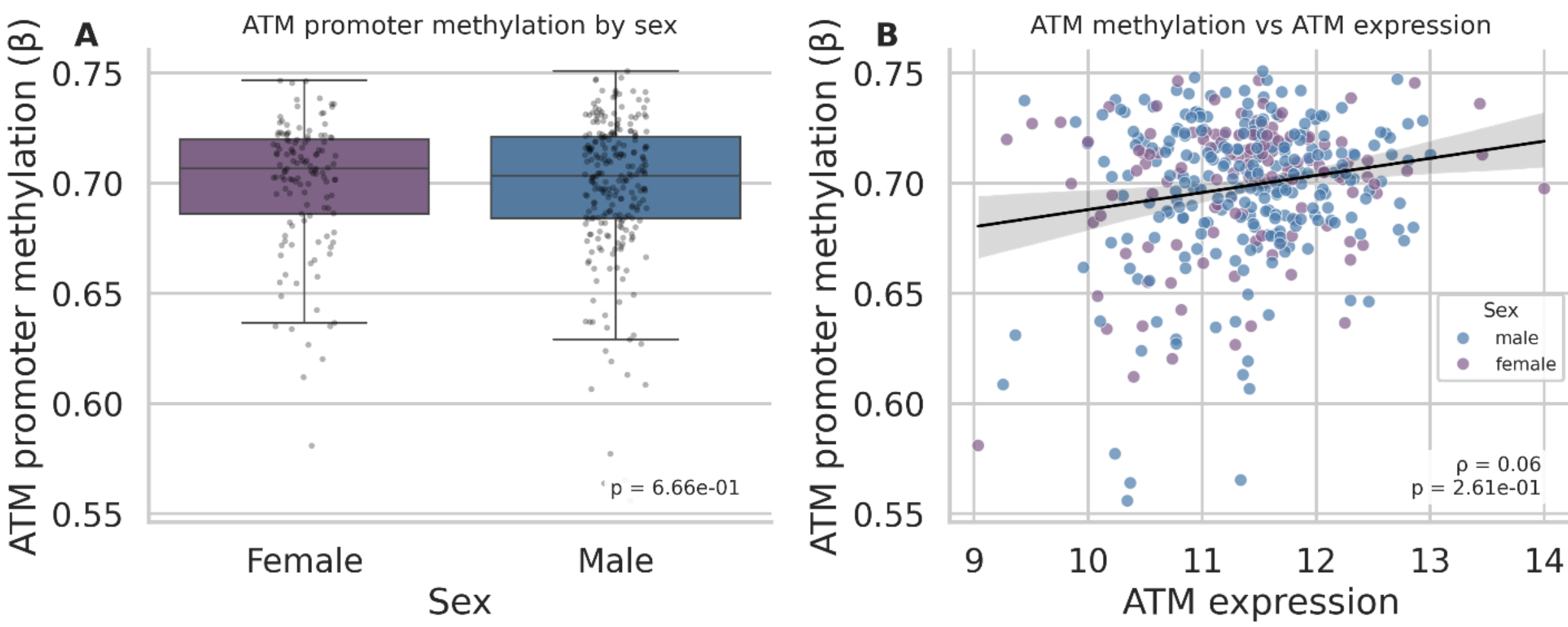

(**A**) ATM promoter methylation levels (β values) stratified by sex. No significant difference in methylation was observed between female and male tumors (Mann–Whitney test $p = 6.66 \times 10^{-1}$). (**B**) Relationship between ATM promoter methylation and ATM expression across tumors. No significant association was observed between methylation and gene expression (Spearman $\rho = 0.06$, $p = 2.61 \times 10^{-1}$). Points are colored by sex.

Fig. S2. Frequency of hypermutated tumors by sex in TCGA-STAD.

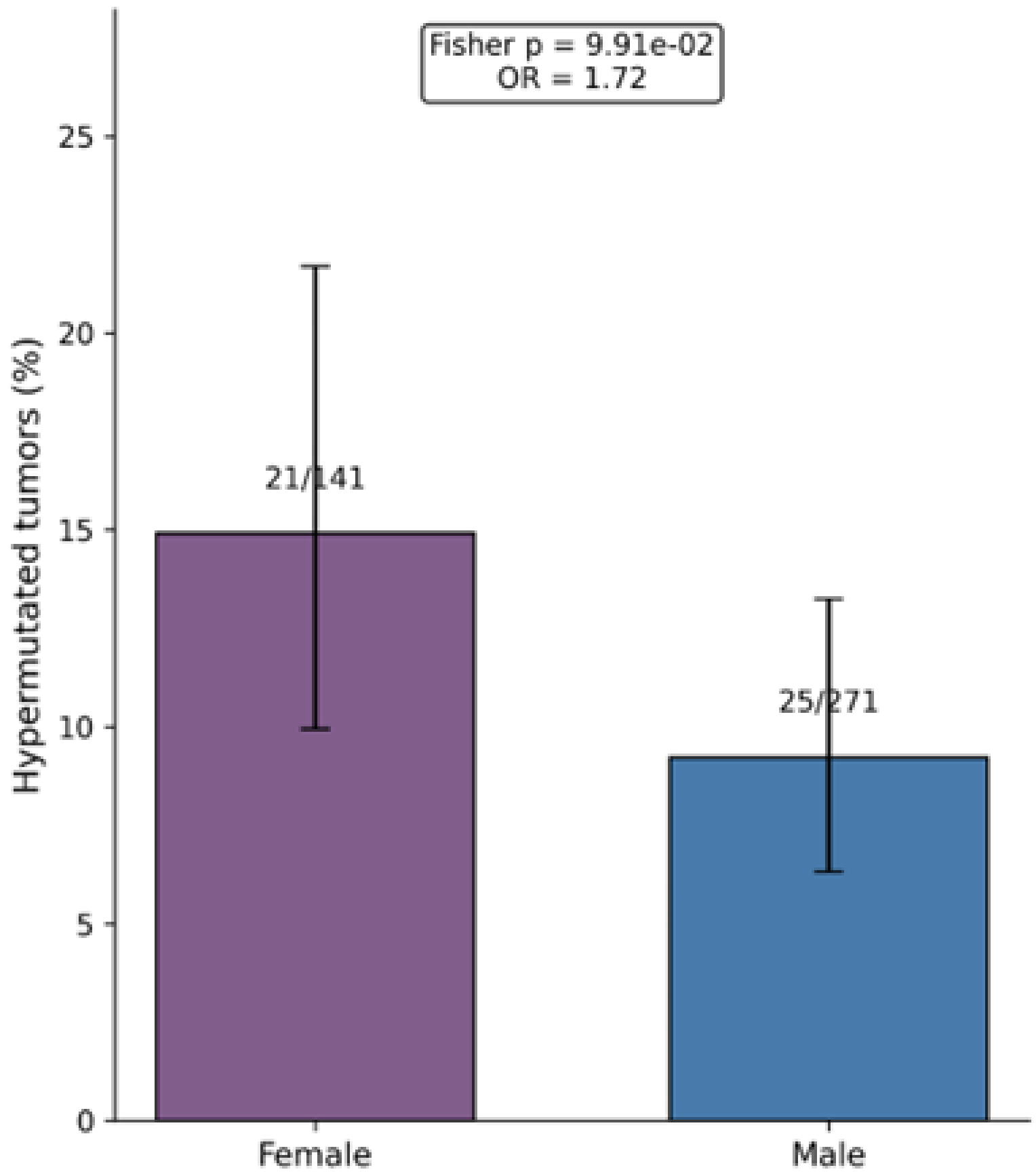

Fraction of hypermutated tumors, defined as tumors with >2000 autosomal nonsynonymous mutations, stratified by sex in TCGA-STAD. Hypermutated tumors were more frequent among female tumors (21/141, 14.9%) than male tumors (25/271, 9.2%), although this difference did not reach statistical significance (Fisher's exact test $p = 0.099$; odds ratio = 1.72). Error bars indicate 95% confidence intervals.

Fig. S3: MSI tumors are characterized by increased MLH1 promoter methylation, reduced MLH1 expression, and elevated mutation burden

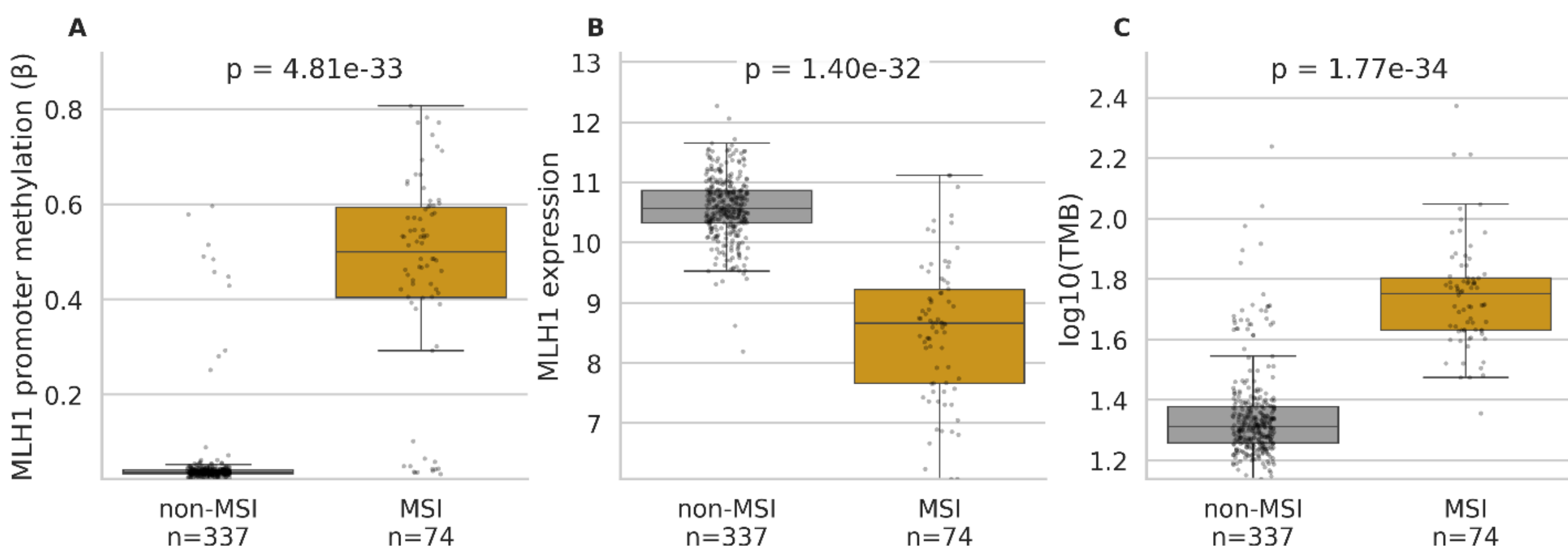

(A) MLH1 promoter methylation levels in MSI and non-MSI tumors. MSI tumors exhibit significantly higher methylation compared with non-MSI tumors (Mann–Whitney test $p = 4.81 \times 10^{-33}$). **(B)** MLH1 expression levels in MSI and non-MSI tumors. MSI tumors show significantly reduced MLH1 expression relative to non-MSI tumors (Mann–Whitney test $p = 1.40 \times 10^{-32}$). **(C)** Tumor mutational burden (log10-transformed TMB) in MSI and non-MSI tumors. MSI tumors exhibit substantially higher mutation burden compared with non-MSI tumors (Mann–Whitney test $p = 1.77 \times 10^{-34}$).

Fig. S4: MLH1 promoter methylation distribution and hypermutation transition

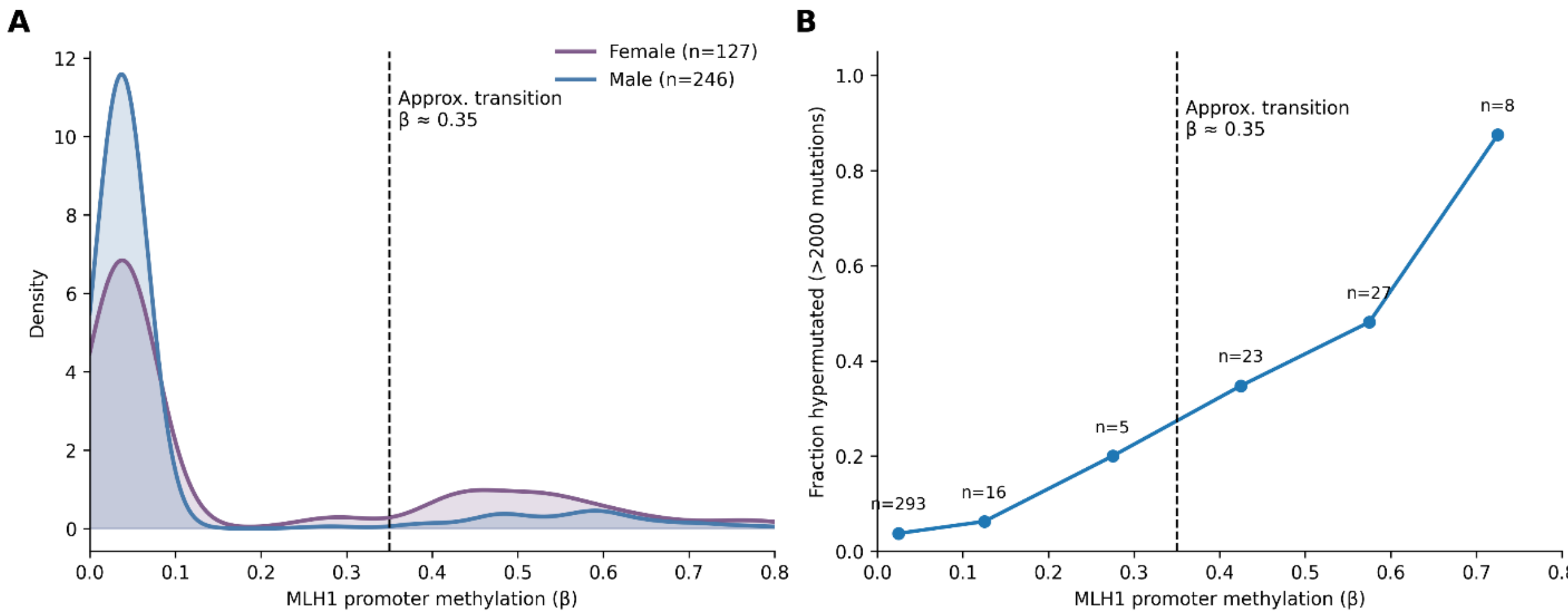

(**A**) Kernel density distribution of MLH1 promoter methylation β values in TCGA-STAD tumors, stratified by sex (female, n = 127; male, n = 246). A vertical dashed line marks an approximate transition point at $\beta \approx 0.35$. Female tumors showed a broader distribution with an extended higher-methylation tail; the underlying sex-stratified methylation distributions differed by Mann–Whitney U test ($p = 5.52 \times 10^{-4}$). KDE curves are shown as descriptive distribution summaries rather than formal tests of peak position. (**B**) Fraction of hypermutated tumors (TMB > 2000 mutations) across increasing MLH1 promoter methylation bins. Each point represents the proportion of hypermutated tumors within a given β interval, with sample size indicated above each point. The fraction of hypermutated tumors increased progressively with higher methylation levels, with an apparent increase above $\beta \approx 0.35$; MLH1 promoter methylation differed across mutation-burden groups by Kruskal–Wallis test ($p = 3.05 \times 10^{-23}$).

Fig. S5: Age distribution of MSI and non-MSI gastric tumors

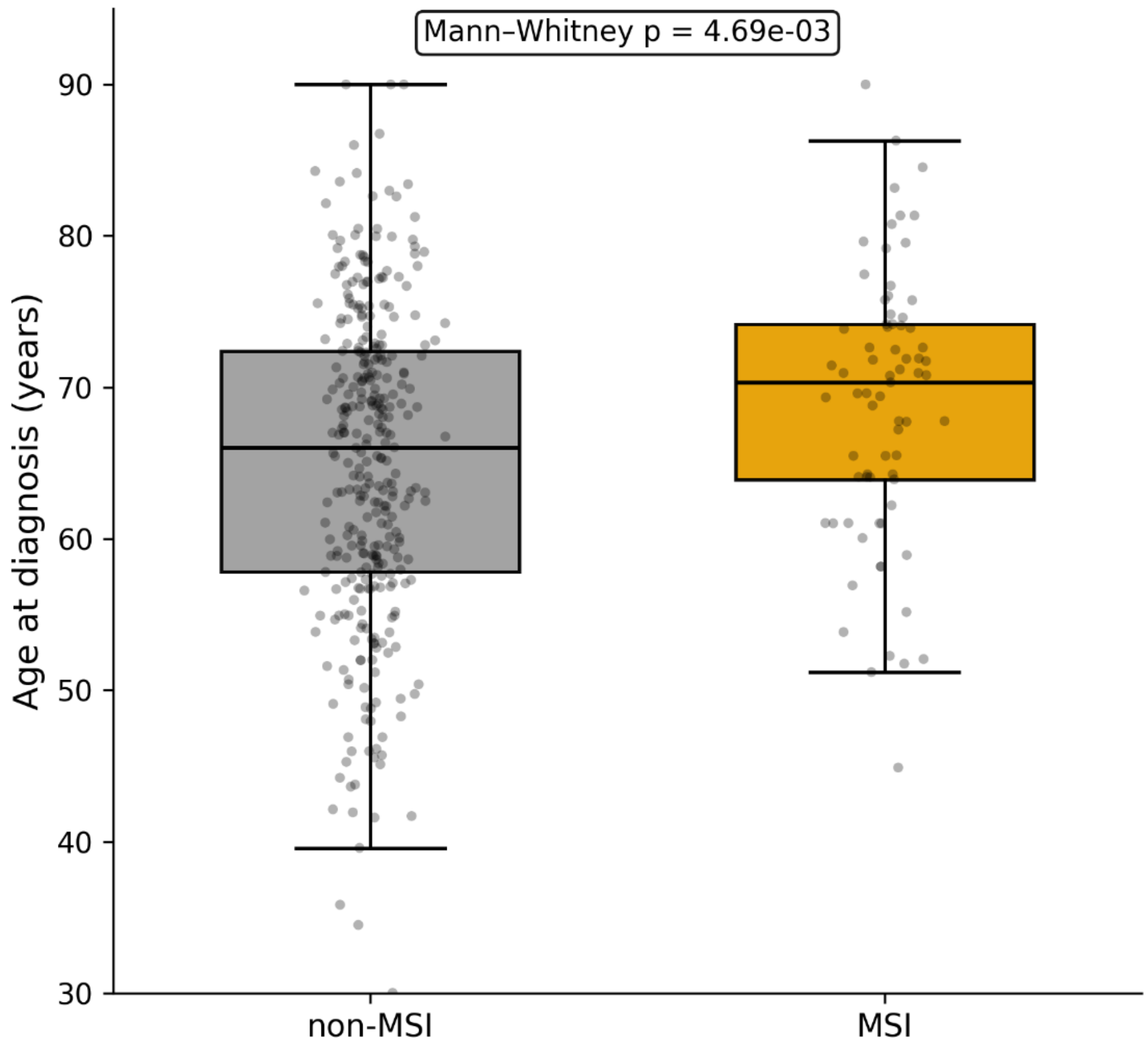

Age at diagnosis for MSI and non-MSI tumors in the TCGA-STAD cohort. MSI tumors occurred in significantly older patients compared with non-MSI tumors (Mann–Whitney $p = 4.69 \times 10^{-3}$). This analysis supports the inclusion of age as a covariate in models evaluating MSI-associated differences.

Fig. S6. Subtype-aware tumor mutational burden analyses in TCGA-STAD.

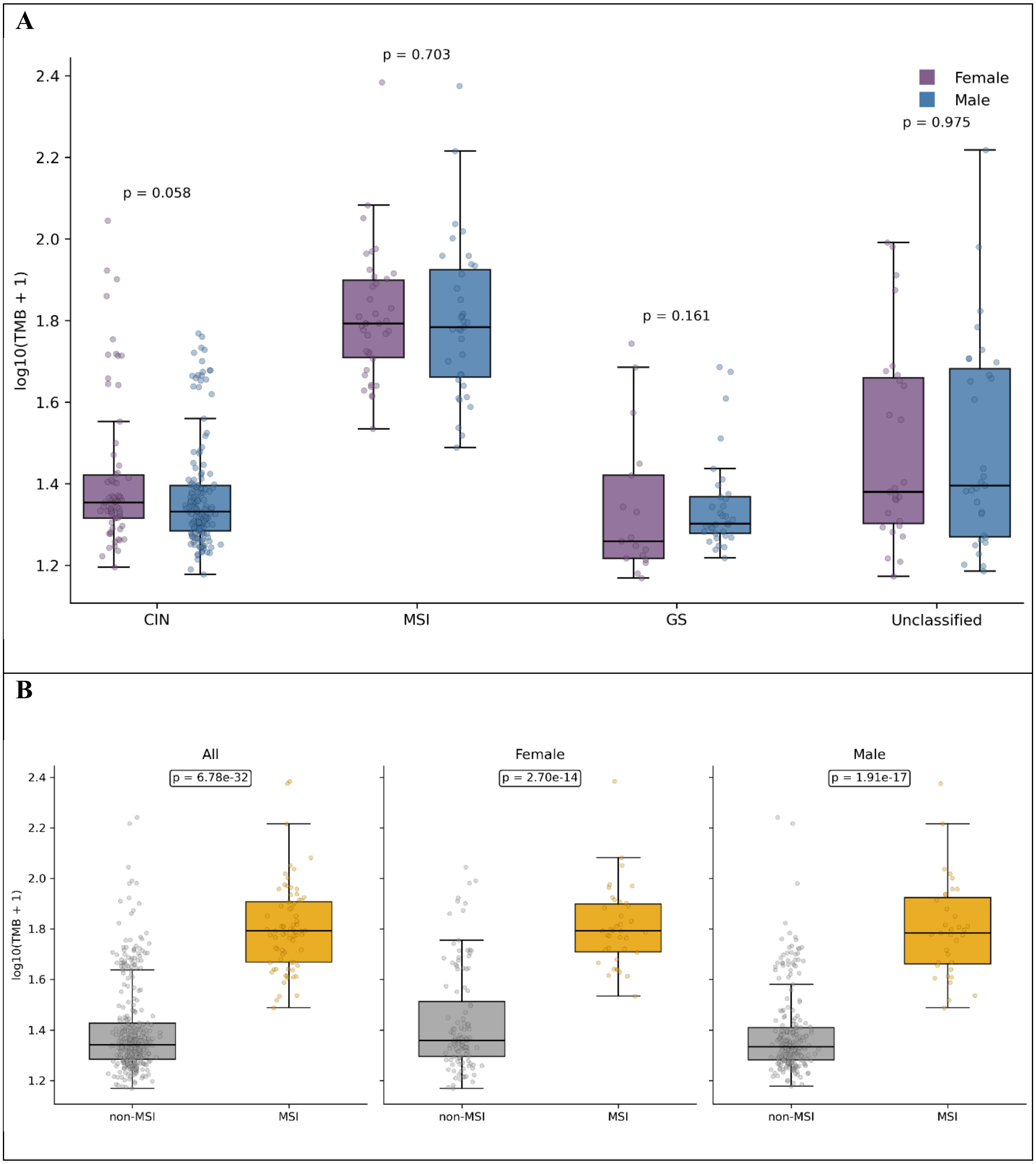

(**A**) Female and male log10(TMB + 1) distributions within TCGA PanCancer molecular subtype groups. Subtypes shown are chromosomal instability (CIN), MSI/hypermutated-indel,

genomically stable (GS), and unclassified tumors. **(B)** Log10(TMB + 1) distributions in MSI and non-MSI tumors, shown for all tumors and separately by sex.

For both panels, boxes indicate the interquartile range, horizontal lines indicate medians, whiskers indicate 1.5× the interquartile range, and points represent individual tumors. P-values indicate two-sided Mann–Whitney U tests.
